# Supplementary material for: Heart‐On‐a‐Chip with Integrated Ultrasoft Mechanosensors for Continuous Measurement of Cell‐ and Tissue‐Scale Contractile Stresses
Source: Small. 2025 Dec 31;22(9):e04493. doi: 10.1002/smll.202504493 (PMC12895230; doi:10.1002/smll.202504493)
Supplement: Supplementary file 1 — Supporting File 1: smll71999‐sup‐0001‐SuppMat.docx. [file SMLL-22-e04493-s002.docx]

**Supporting Information**

**Heart-On-a-Chip with Integrated Ultrasoft Mechanosensors for Continuous Measurement of Cell- and Tissue-scale Contractile Stresses**

Ali Mousavi^a,b,c^, Christina-Marie Boghdady^d^, Shihao Cui^b^, Sabra Rostami^d^, Amid Shakeri^e^, Naimeh Rafatian^e^, Mark Aurousseau^f^, Gregor Andelfinger^b^, Milica Radisic^e,g^, Christopher Moraes^d^, and Houman Savoji ^a,b,c,h,i*^

^a^ Institute of Biomedical Engineering, Department of Pharmacology and Physiology, Faculty of Medicine, Université de Montréal, Montréal, QC, Canada

^b^ CHU Sainte-Justine Research Center, Montréal, QC, Canada

^c^ Montréal TransMedTech Institute, Montréal, QC, Canada

^d^ Department of Chemical Engineering, McGill University, Montréal, QC, Canada

^e^ Institute of Biomedical Engineering, University of Toronto, Toronto, ON, Canada

^f^ eNUVIO Inc., Montréal, QC, Canada

^g^ Terrence Donnelly Centre for Cellular & Biomolecular Research, University of Toronto, Toronto, ON, Canada

^h^ Department of Mechanical Engineering, Polytechnique Montréal, Montréal, QC, Canada

^i^ Institute of Biomedical Engineering, Polytechnique Montréal, Montréal, QC, Canada

*** Corresponding Author**

**Houman Savoji** - Department of Mechanical Engineering, Polytechnique Montréal, Montréal, QC, Canada, Institute of Biomedical Engineering, Polytechnique Montréal, Montréal, QC, Canada, Institute of Biomedical Engineering, Department of Pharmacology and Physiology, Faculty of Medicine, University of Montréal, Montréal, QC, Canada; CHU Sainte-Justine Research Center, Montréal, QC, Canada; Montréal TransMedTech Institute (iTMT), Montréal, QC, Canada

E-mail: [h.savoji@polymtl.ca](mailto:h.savoji@polymtl.ca)

**Supplementary Figures**


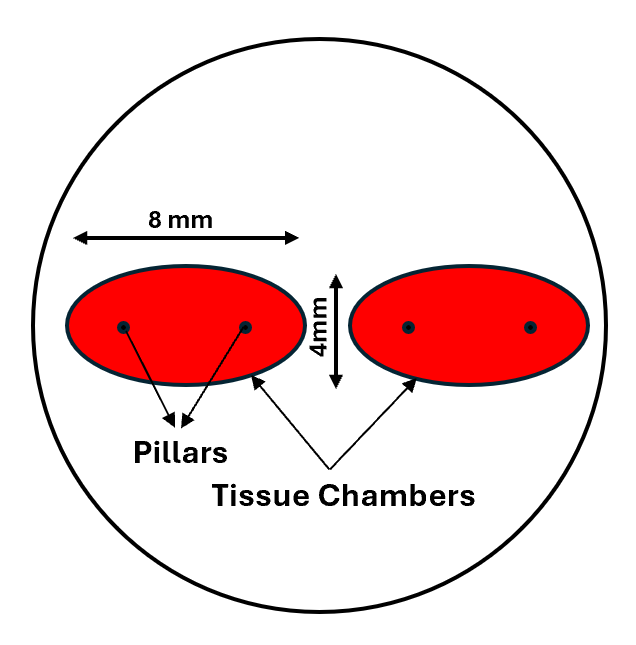


**Fig. S1.** Schematics of chip design and the dimensions.


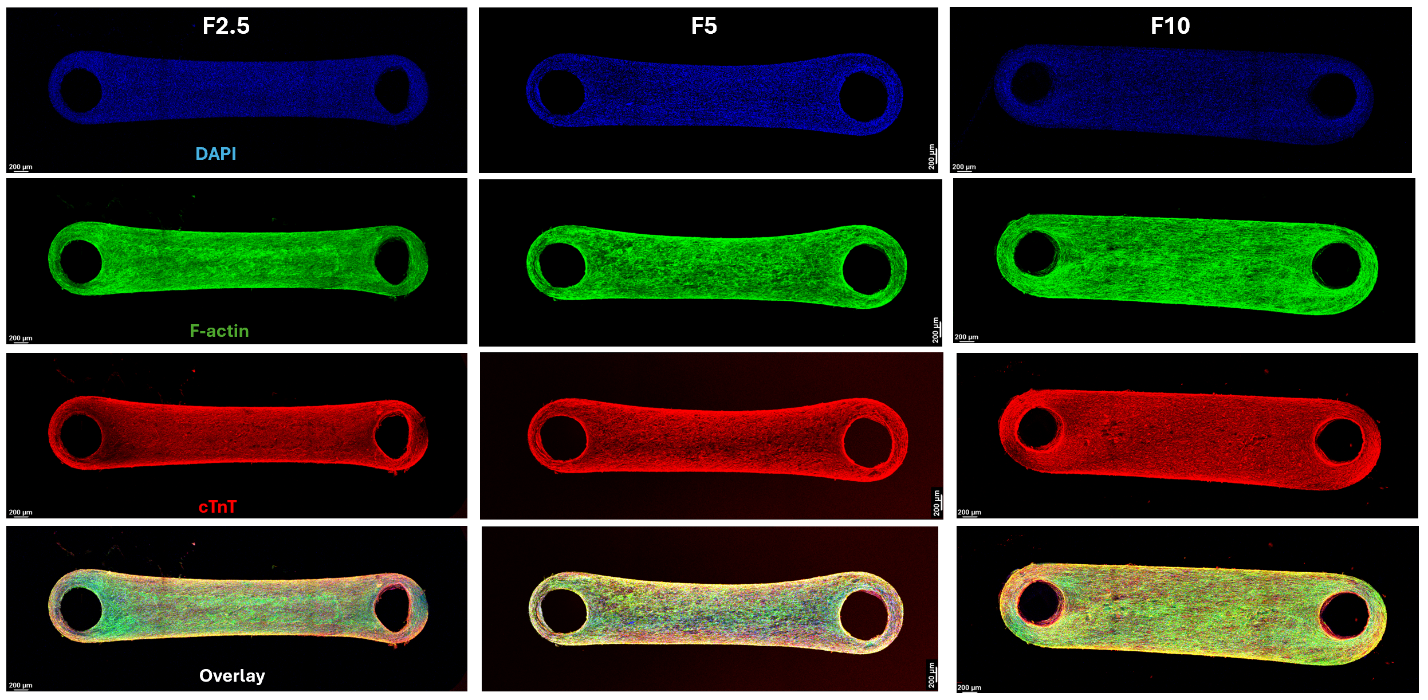


**Fig. S2.** Different channels of confocal imaging for the whole mount IF staining of F2.5, F5, and F10 cardiac microtissues after 7 days of culture (blue channel: DAPI, red channel: cTnT, green channel: phalloidin) and overlay images. Scale bar = 200 µm.


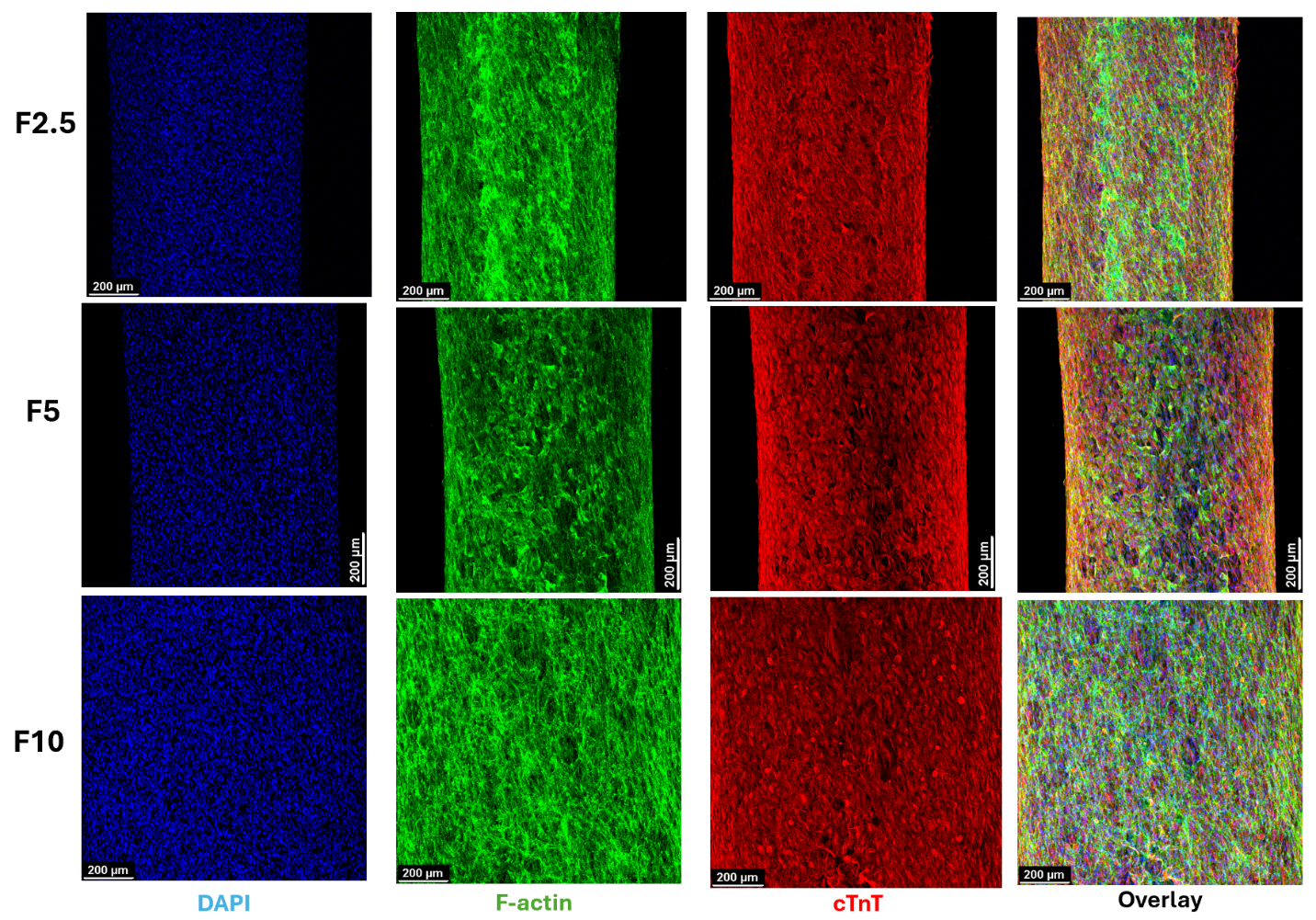


**Fig. S3.** Different channels of confocal imaging for the IF staining of F2.5, F5, and F10 cardiac microtissues (10x objective) after 7 days of culture (blue channel: DAPI, red channel: cTnT, green channel: phalloidin) and overlay images. Scale bar = 200 µm.


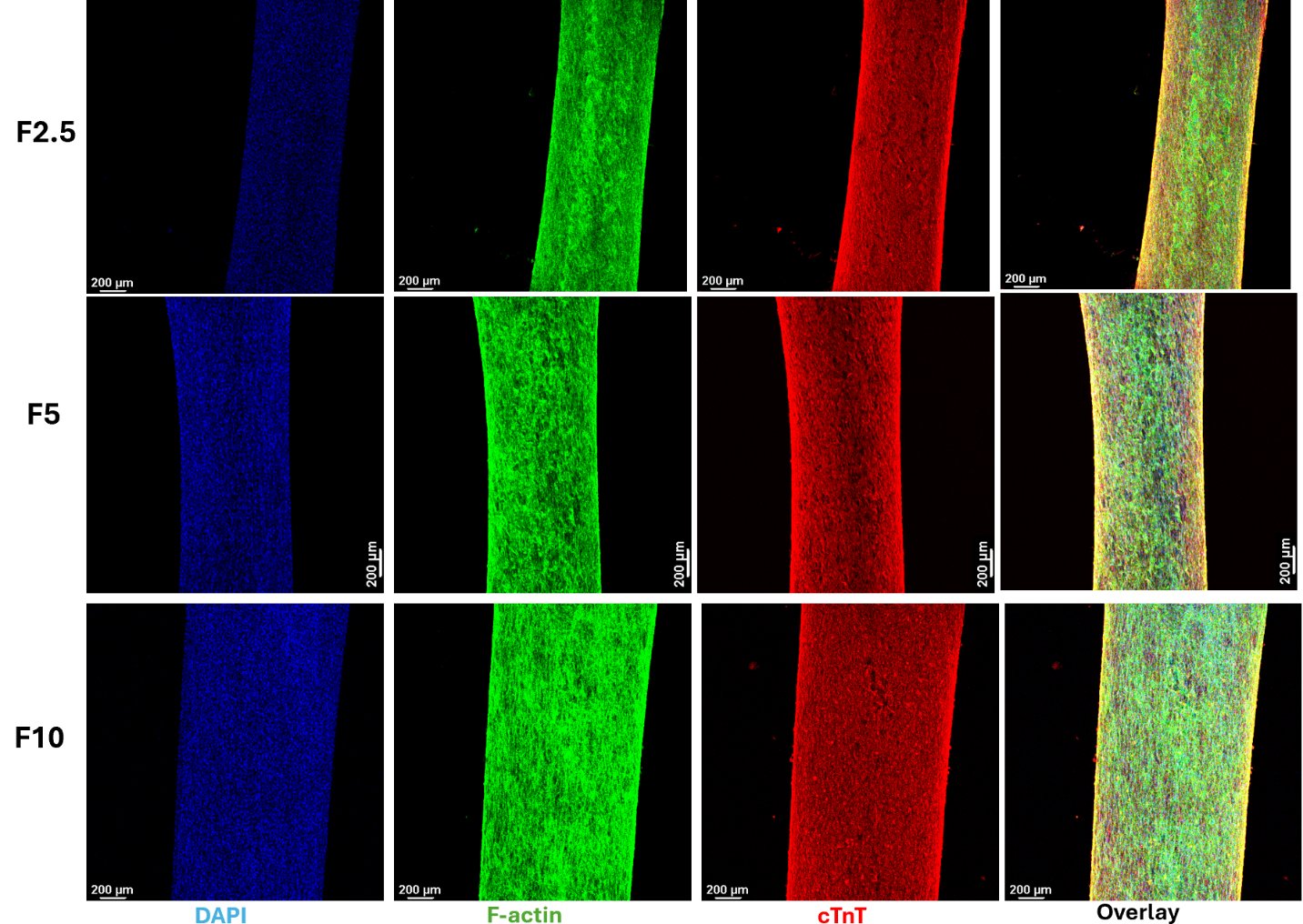


**Fig. S4.** Different channels of confocal imaging for the IF staining of F2.5, F5, and F10 cardiac microtissues (5x objective) after 7 days of culture (blue channel: DAPI, red channel: cTnT, green channel: phalloidin) and overlay images. Scale bar = 200 µm.


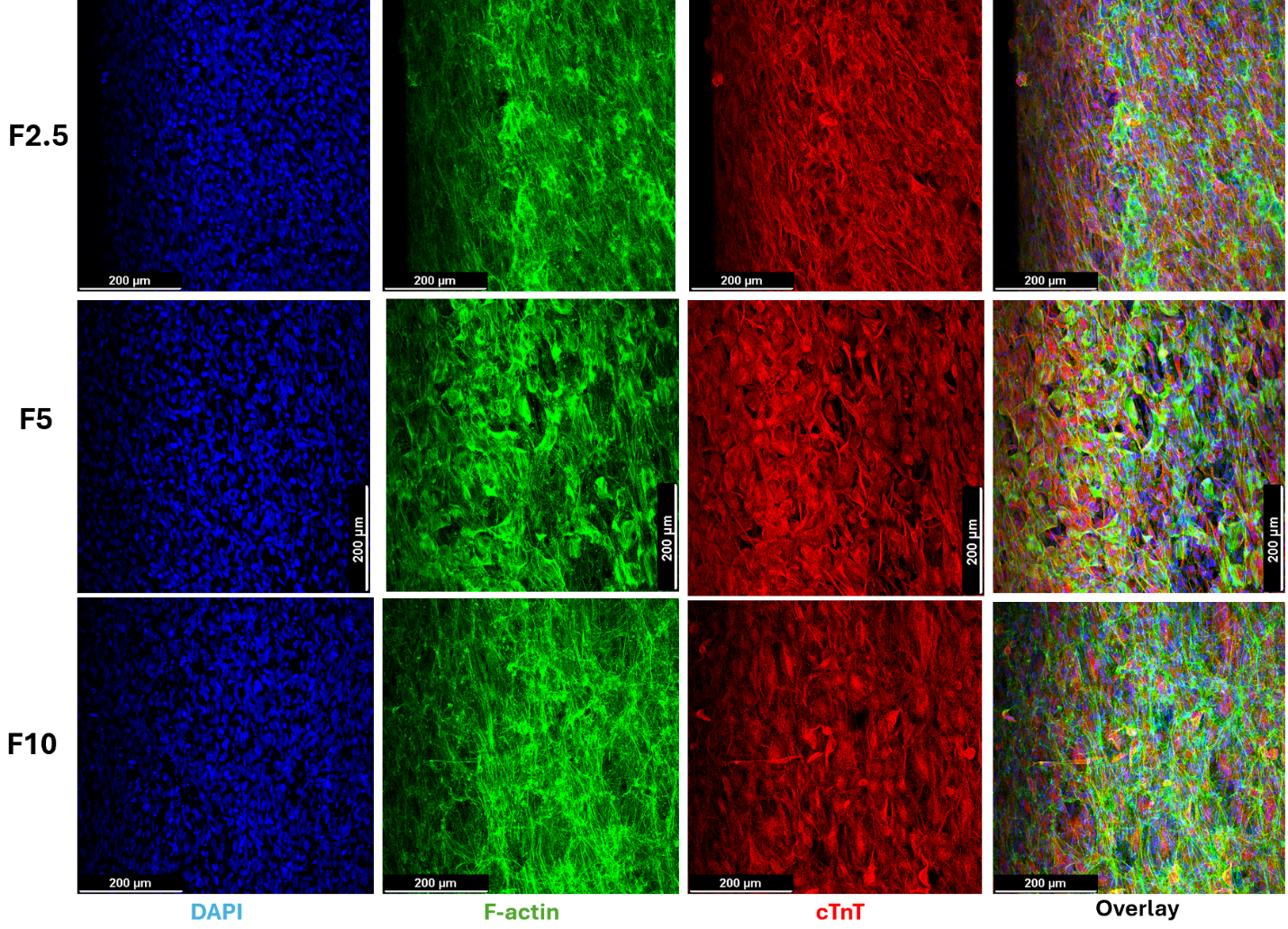


**Fig. S5.** Different channels of confocal imaging for the IF staining of F2.5, F5, and F10 cardiac microtissues (20x objective) after 7 days of culture (blue channel: DAPI, red channel: cTnT, green channel: phalloidin) and overlay images. Scale bar = 200 µm.


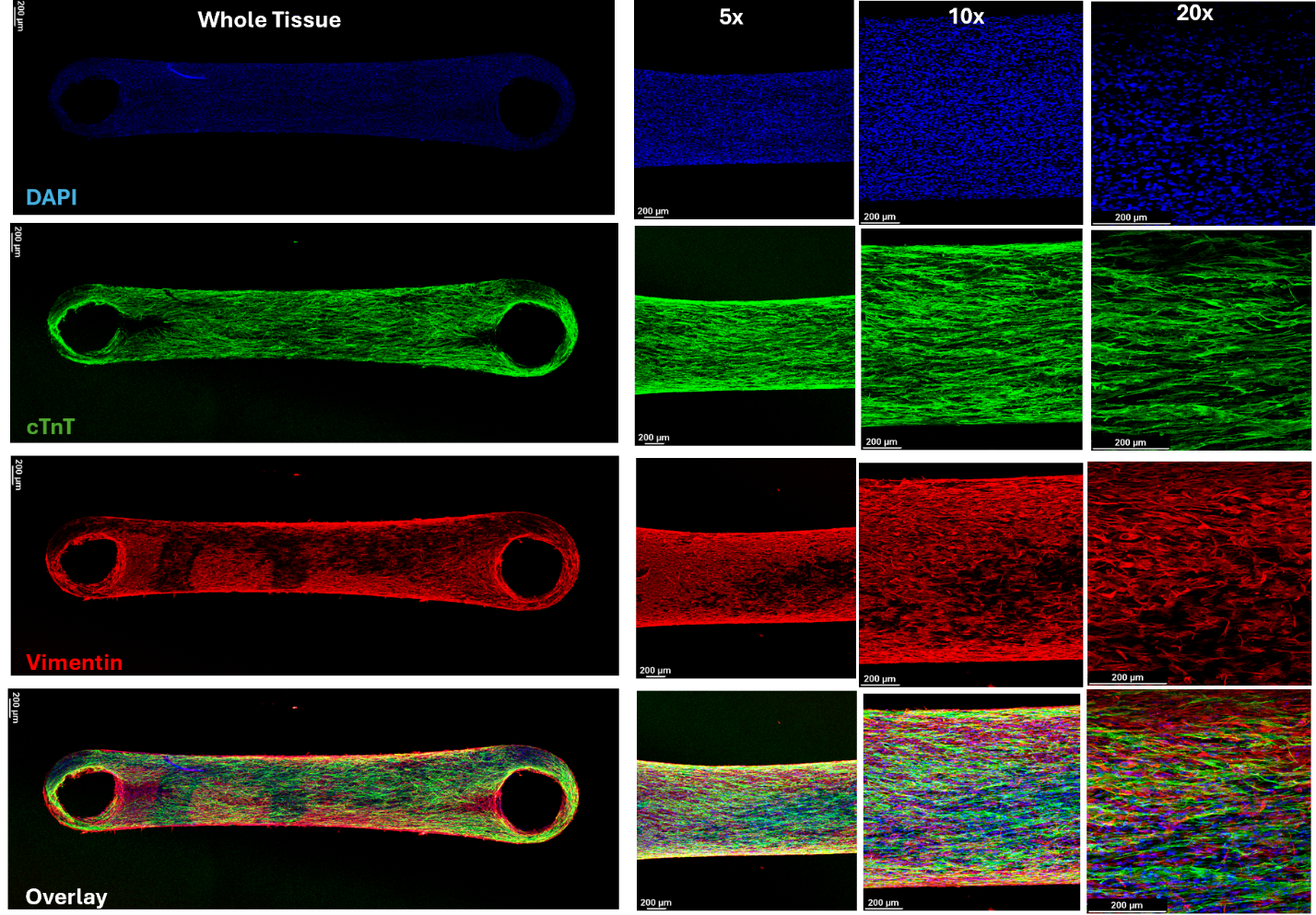


**Fig. S6.** Different channels of confocal imaging for the whole mount IF staining of cardiac microtissues for CMs vs non-CMs as well as different resolutions (5x, 10x, and 20x) after 7 days of culture (blue channel: DAPI, red channel: vimentin, green channel: cTnT) and overlay images. Scale bar = 200 µm.


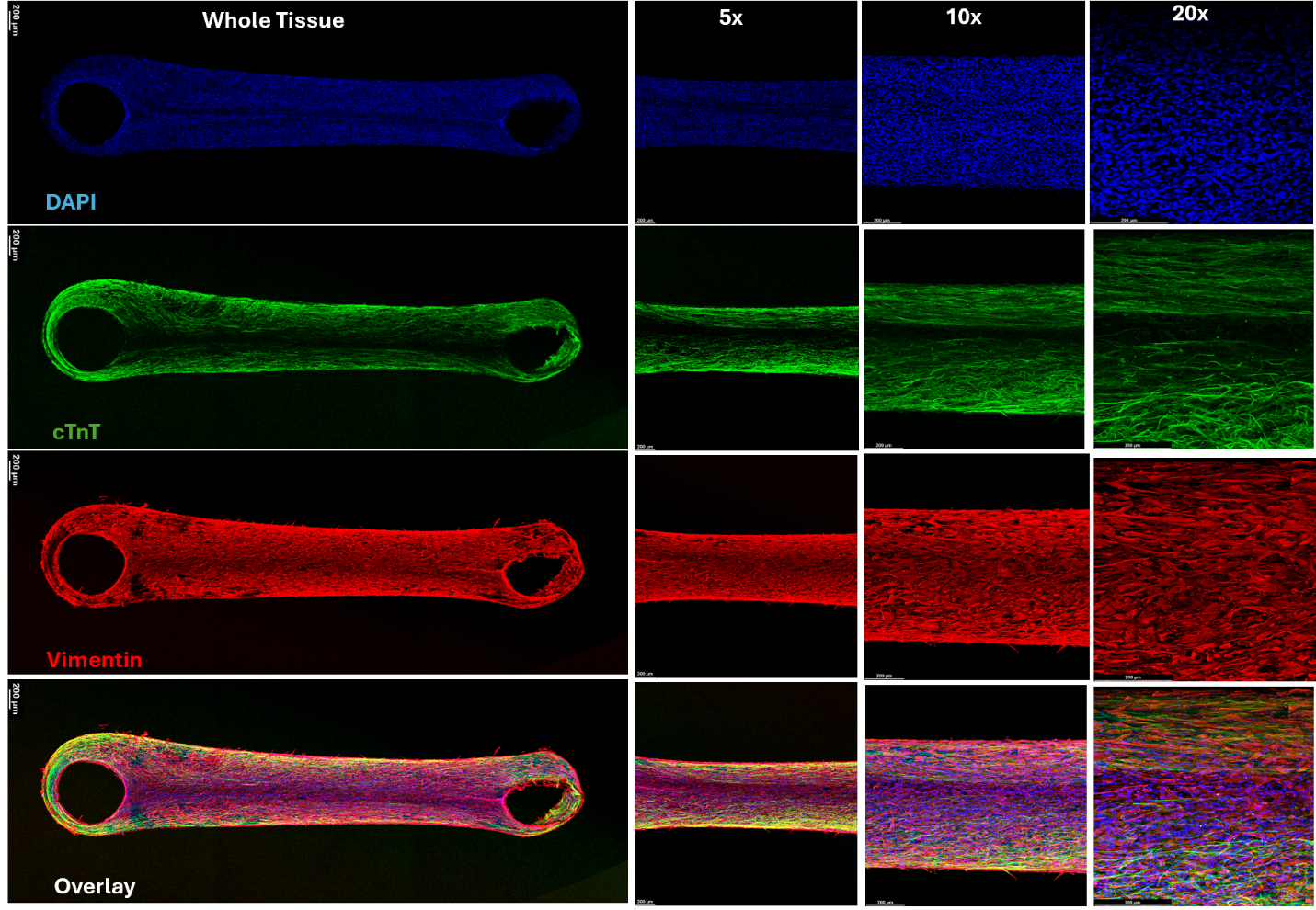


**Fig. S7.** Different channels of confocal imaging for the whole mount IF staining of cardiac microtissues for CMs vs non-CMs as well as different resolutions (5x, 10x, and 20x) after 14 days of culture (blue channel: DAPI, red channel: vimentin, green channel: cTnT) and overlay images. Scale bar = 200 µm.


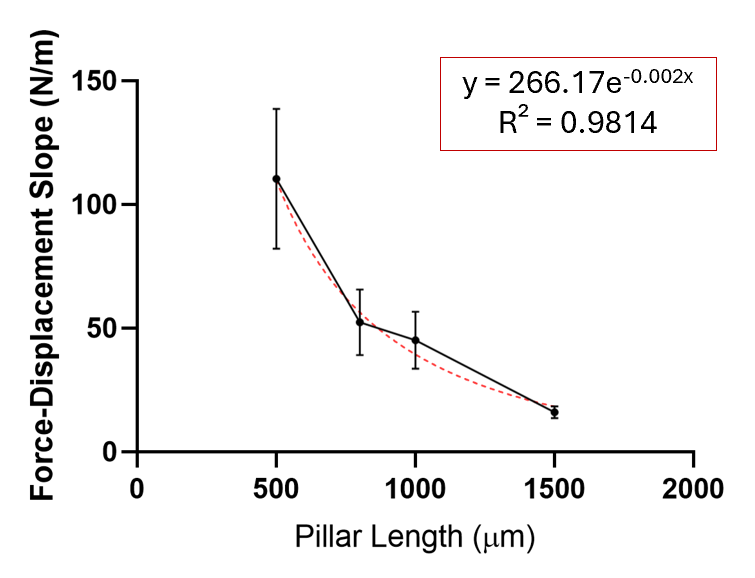


**Fig. S8.** Relationship between slop of force-displacement curve and pillar length (in the testing range of 0.5 to 1.5 mm).


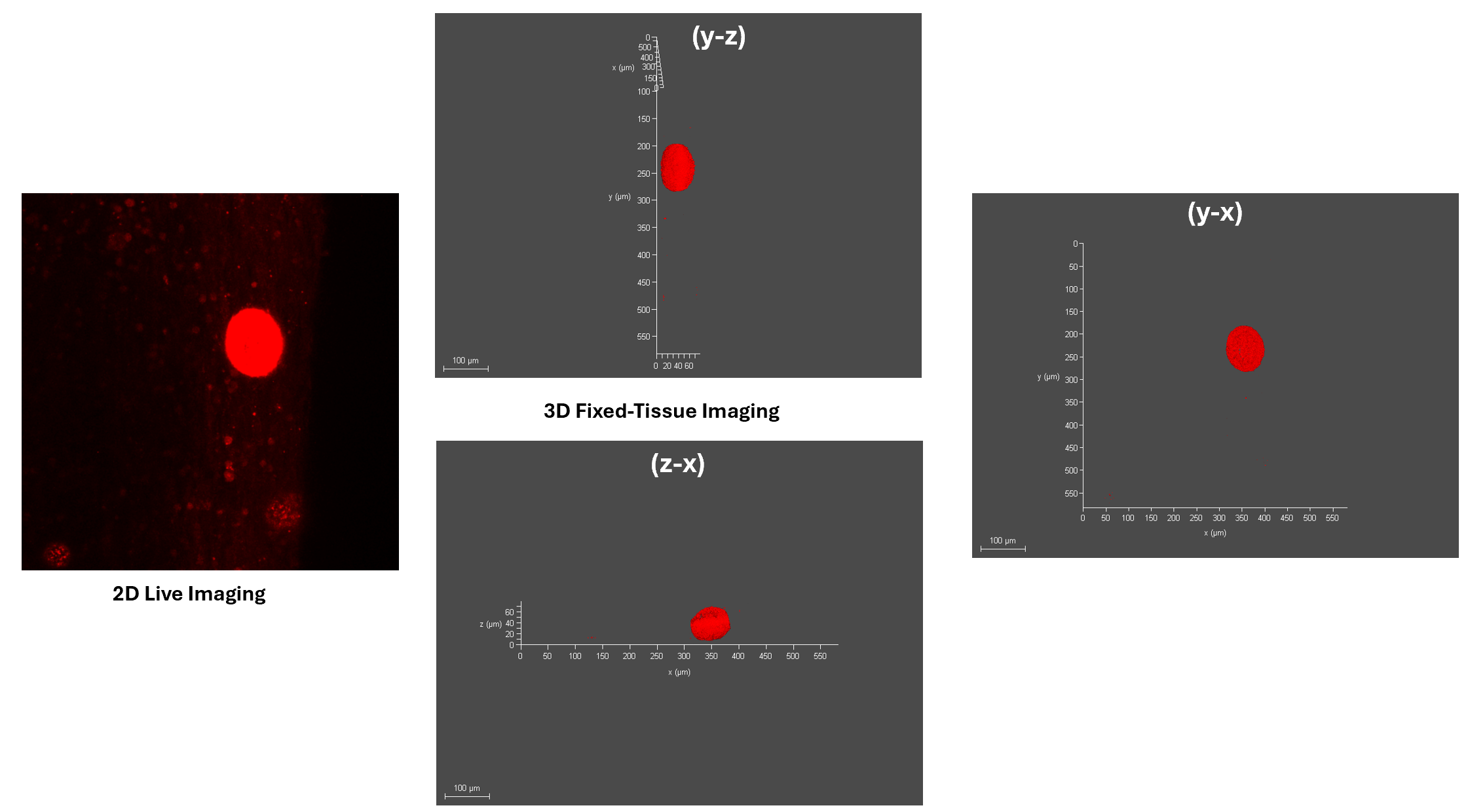


**Fig. S9.** Representative images of eMSGs before fixation (2D live imaging) and after fixation (3D confocal imaging) in y-z, z-x, and y-x orientations. Scale bar=100μm.


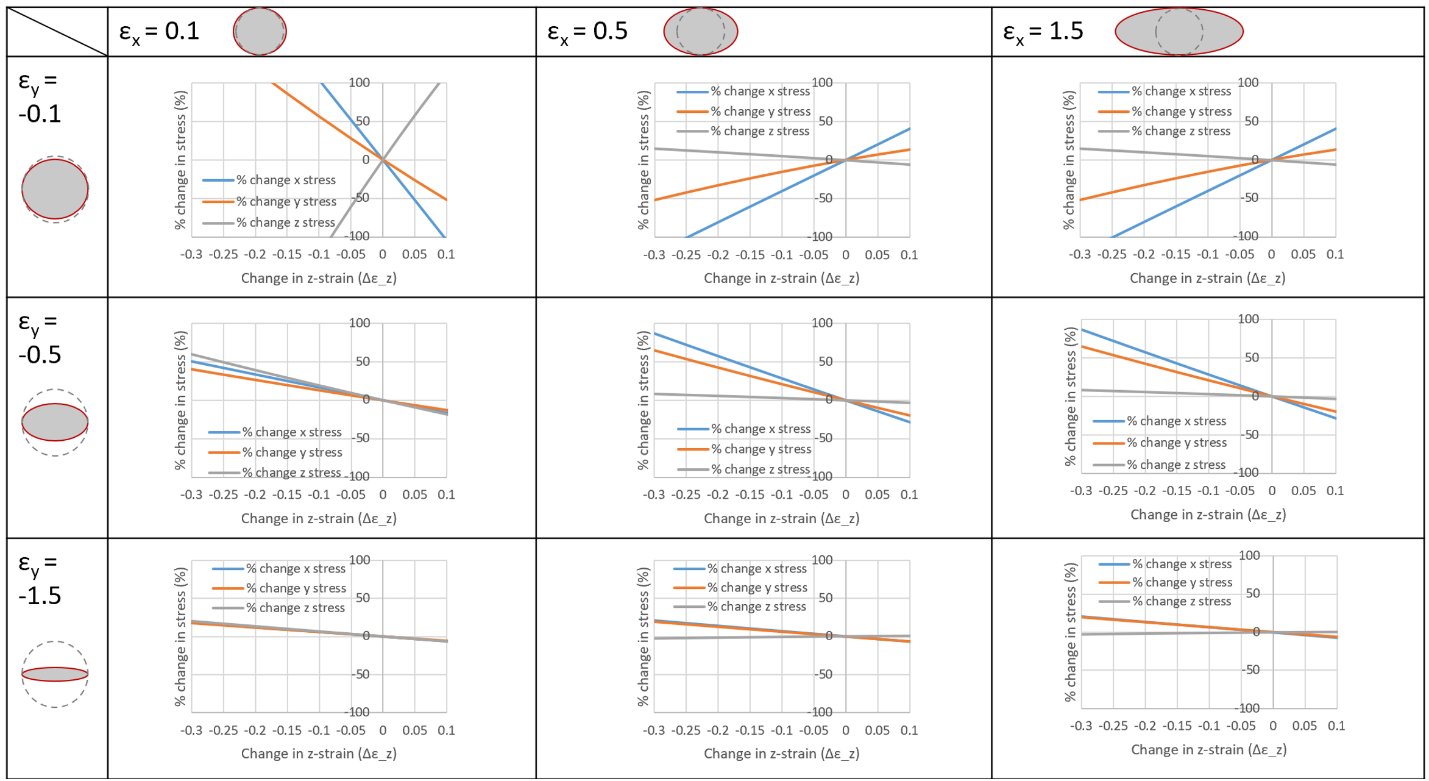


**Fig. S10.** COMSOL 3D modeling of the system with different strains in x and y orientations to estimate the error percentage of 2D stress measurements.


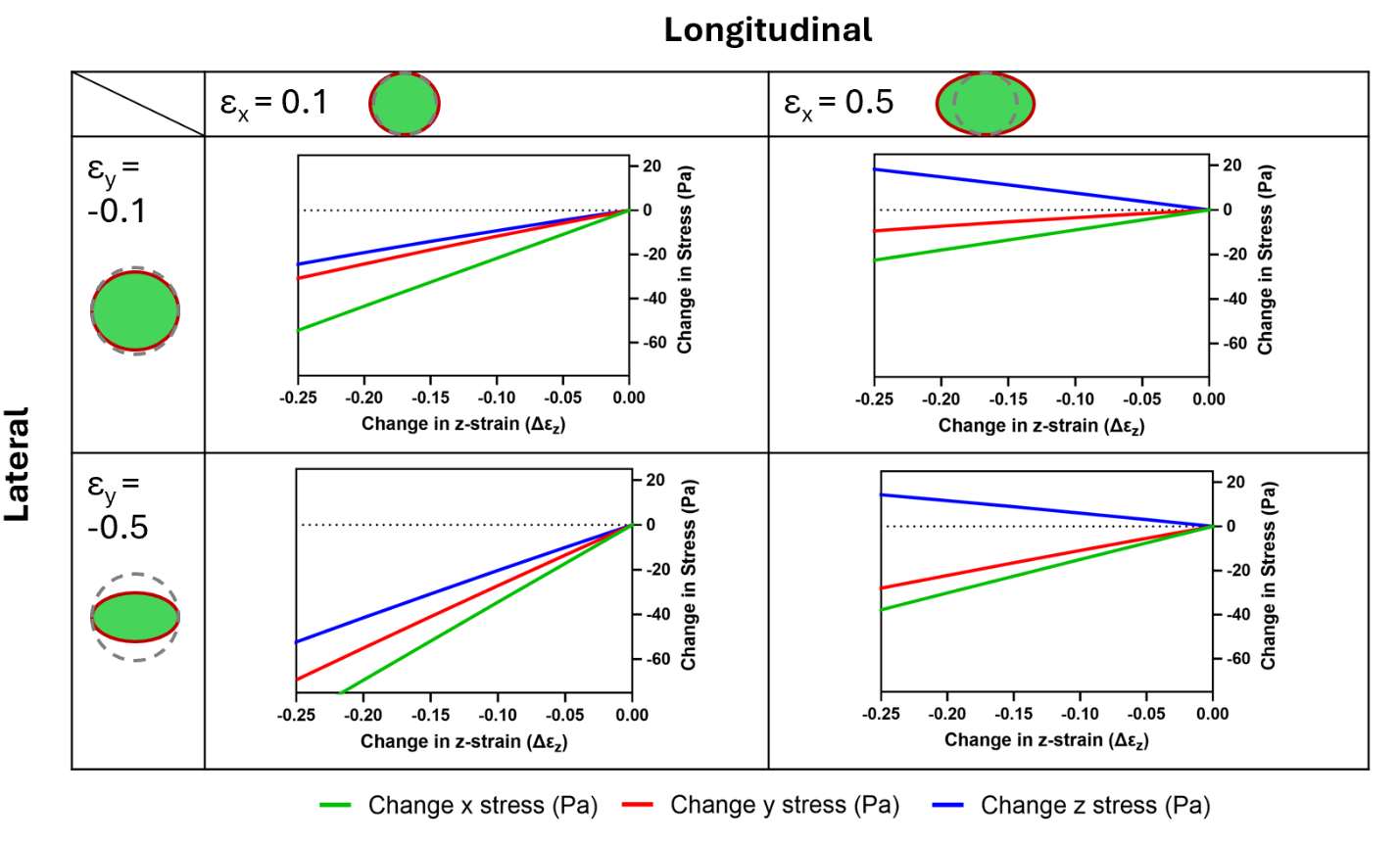


**Fig. S11.** COMSOL 3D modeling of the system to estimate the actual change in stresses (Pa) based on strain values in the x and y directions.

**Supplementary Movies**

**Movie S1:** Microsquisher analysis of pillars at 500, 800, 1000, and 1500 µm heights from the base.

**Movie S2:** Spontaneous beating of F2.5 cardiac microtissues on day 7 of culture.

**Movie S3:** Spontaneous beating of F5 cardiac microtissues on day 7 of culture.

**Movie S4:** Spontaneous beating of F10 cardiac microtissues on day 7 of culture.

**Movie S5:** Calcium transients of F5 cardiac microtissues on day 7 of culture.

**Movie S6:** Calcium transients of F10 cardiac microtissues on day 7 of culture.

**Movie S7:** Calcium transients of F5 cardiac microtissues on day 14 of culture.

**Movie S8:** Spontaneous beating of EHTs before the drug treatment (CTRL group).

**Movie S9:** Spontaneous beating of EHTs after the treatment with norepinephrine (NE group).

**Movie S10:** 3D view of a representative sensor by confocal imaging after tissue fixation.

**Movie S11:** A representative video showing sensor movement during tissue contraction.

**Movie S12:** Image processing of Movie S11 to identify sensor deformation during contraction, including image stabilization, noise reduction, and contrast adjustment

**Movie S13:** Image processing of Movie S11 to identify sensor deformation during contraction, including background subtraction and segmentation, followed by edge detection and thresholding

**Movie S14:** Image processing of Movie S11 to identify sensor deformation during contraction, including optical flow analysis and the application of tracking algorithms to quantify motion between frames.
