# Supplementary figures and images for: Heart‐On‐a‐Chip with Integrated Ultrasoft Mechanosensors for Continuous Measurement of Cell‐ and Tissue‐Scale Contractile Stresses
Source: Small. 2025 Dec 31;22(9):e04493. doi: 10.1002/smll.202504493 (PMC12895230; doi:10.1002/smll.202504493)

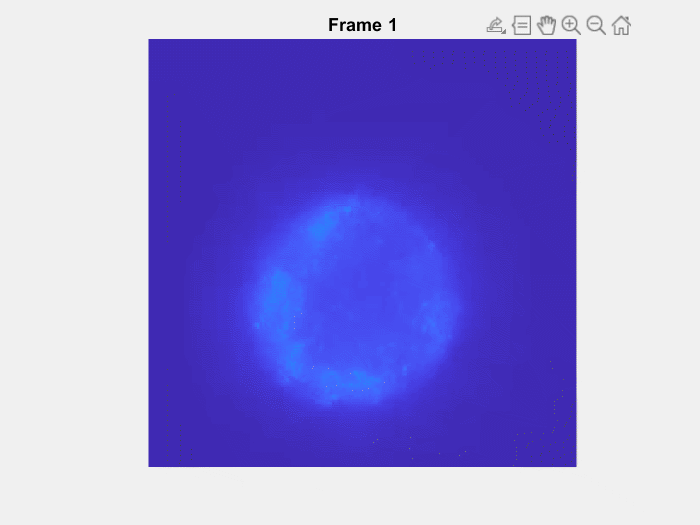

Supplement: Supplementary file 13 — Supporting File 13: smll71999‐sup‐0013‐MovieS12.gif. [file SMLL-22-e04493-s012.gif]

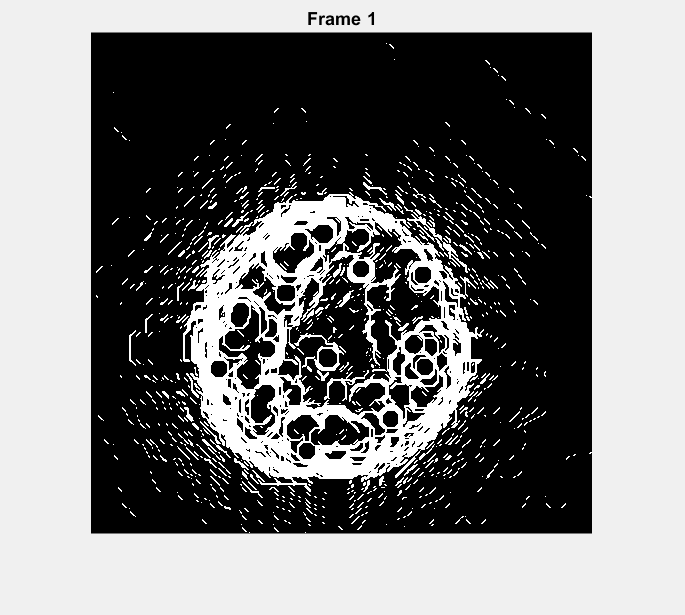

Supplement: Supplementary file 14 — Supporting File 14: smll71999‐sup‐0014‐MovieS13.gif. [file SMLL-22-e04493-s013.gif]

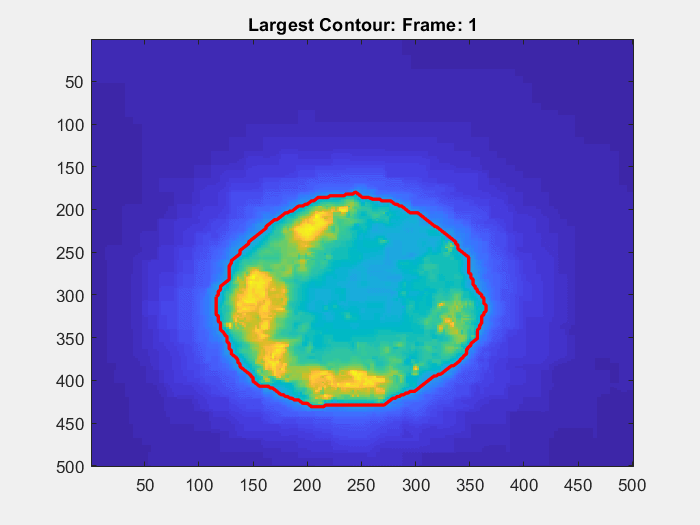

Supplement: Supplementary file 15 — Supporting File 15: smll71999‐sup‐0015‐MovieS14.gif. [file SMLL-22-e04493-s015.gif]
